# Diagnosis and treatment of a boy with IPEX syndrome presenting with diabetes in early infancy

**DOI:** 10.1002/ccr3.2438

**Published:** 2019-09-27

**Authors:** Sejal Kadakia, Lauge Farnaes, David Dimmock, Shimul Chowdhury, Yan Ding, Eric J. Anderson, Stephen Kingsmore, Ron S. Newfield

**Affiliations:** ^1^ Division of Pediatric Endocrinology Children's Hospital of Orange County Orange CA USA; ^2^ Rady Children's Hospital of San Diego San Diego CA USA; ^3^ Rady Children's Institute for Genomic Medicine San Diego CA USA; ^4^ Division of Pediatric Hematology‐Oncology University of California San Diego San Diego CA USA; ^5^ Division of Pediatric Endocrinology University of California San Diego San Diego CA USA

**Keywords:** autoimmunity, bone marrow transplantation, diabetes mellitus, infant, X‐linked

## Abstract

IPEX syndrome (Immune dysregulation, Polyendocrinopathy, X‐linked) should be tested for in males under 6 months old presenting with diabetes, even without other IPEX features. Early diagnosis and bone marrow transplantation can improve outcomes.

## Graphical abstract

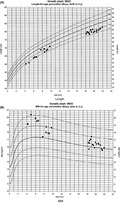

## 1 INTRODUCTION

Neonatal diabetes is a rare and genetically heterogeneous disease seen in 1:100 00 infants or less, typically diagnosed prior to age 6 months. There are more than 20 monogenic causes of neonatal diabetes, in addition to methylation abnormalities at the 6q24 locus.^1^ Unlike type 1 diabetes mellitus, the etiology of neonatal diabetes is typically not autoimmune. Neonatal diabetes can be transient, isolated permanent, or associated with other features (eg, skeletal dysplasia and liver disease in the autosomal recessive Wolcott‐Rallison syndrome, due to *EIF2AK3* mutations). Over 40% of cases in nonconsanguineous families are due to potassium channel gene mutations (*KCNJ11* or *ABCC8*), whereas in infants born to consanguineous parents, the most common cause (24%) is a homozygous mutation in the *EIF2AK3* gene.^1^ IPEX syndrome (Immune dysregulation, Polyendocrinopathy, X‐linked) is an extremely rare genetic disorder associated with mutations within *FOXP3*, which codes for a forkhead transcription factor that controls the development and function of CD4 + CD25 + regulatory T cells. The prevalence is estimated to be 1 in 1.6 million, and only about 150 cases have been reported to date.^2^ One group reported that 4% of male patients with permanent neonatal diabetes were found to have *FOXP3* mutations.^3^ Mutations in *FOXP3* lead to a severe deficiency or absence of T regulatory cells leading to multiorgan autoimmunity. Unlike other forms of neonatal diabetes, the etiology of neonatal diabetes in IPEX is autoimmune. Other key features of this disorder are enteropathy, dermatitis, failure to thrive, hematologic dysfunction, and autoimmune thyroiditis.^4^ Enteropathy and dermatitis often precede type 1 diabetes, but there are reports of diabetes being the first manifestation.^5^ Here, we report the case of IPEX syndrome in a male infant that presented initially with type 1 diabetes mellitus, without other typical features of IPEX, and discuss the importance of early genetic testing and treatment.

## 2 METHODS

We obtained parental consent to publish this case.

### 2.1 Molecular genetics and analysis

#### 2.1.1 gDNA extraction and library construction

Genomic DNA (gDNA) was isolated from 350 µL EDTA patient blood using QIAGEN EZ1 DSP DNA Blood kit (QIAGEN) on QIAGEN EZ1 Advanced XL system.^6^ The extracted gDNA sample was quantified with Quant‐iT™ dsDNA PicoGreen™ Assay Kit (Thermo Fisher) using SpectraMax^®^ Gemini EM Microplate Spectrofluorometer (Molecular Devices). The integrity of gDNA was confirmed by running 0.8% E‐gel (Thermo Fisher). A DNA library construction started with fragmentation using Covaris Sonicator E220 (Covaris) to shear 1 µg gDNA to the fragments size of 300‐500 base pairs (bp). A fragmented DNA sample was then treated with Illumina TruSeq^®^DNA PCR‐Free Library Prep Kits in order to yield a final library, in which fragments were ligated with a molecular barcoded adapter. The ligated fragment size and concentration of a DNA library sample was measured using Fragment Analyzer™ (Advanced Analytical) and KAPA Library Quantification Kits on Roche LightCycler^®^ 480 Instrument (Roche). A library with the concentration more than 3 nmol/L (300‐600 bp) was passed for sequencing on Novaseq 6000 S2 Flow Cell (FC).

#### 2.1.2 Whole‐genome sequencing (WGS) on novaseq 6000

Six WGS libraries were normalized to obtain a final concentration of 2 nmol/L. 0.5%‐1% Phix library was spiked into the library pool as a positive control. The normalized library in a pool was incubated using 0.2 N sodium hydroxyl (NaOH) at room temperature for 8 minutes in order to denature double‐stranded DNA fragments into single‐stranded ones. 400 mmol/L Tris‐HCl was then used to terminate the denaturing process. The denatured library with a final loading concentration of 300 pmol/L in a pool was then loaded on the S2 FC with the following setting on Illumina Novaseq 6000: S2 FC, Single Index, Read 1:101, Read 2:101, and Index_Read1: 8.

#### 2.1.3 WGS data analysis

WGS data analysis was divided into four phases: primary analysis performed on a sequencer by Illumina Real‐Time Analysis (RTA3) software (Illumina); secondary analysis including mapping or alignment to human reference genome and variant calling (VCF) through Edico Dragon processor (Illumina/Edico Genome); tertiary analysis started with uploading VCF to Opal interface (FABRIC GENOMICS™) to rapidly and accurately prioritize candidate variants by combining deep phenotype data with genomic data using proven proprietary algorithms, and the last phase, that is, the interpretation analysis was manually conducted by qualified personnel. Rapid trio WGS identified a previously described hemizygous, pathogenic canonical splice site variant (c.−23 + 1G > T) in *FOXP3* from these patients’ samples. Post a candidate variant was identified and classified according to The American College of Medical Genetics and Genomics (ACMG) guidelines, a secondary or orthologue test (Sanger sequencing here for single‐nucleotide variations or small insertions or deletions) was ordered and performed in order to confirm a pathogenic or likely pathogenic variant of interest.

#### 2.1.4 Sanger sequencing

A customer designed M13‐tailed forward primer (F) and a reverse primer (R) sequences shown here in italics (F: *TGTAAAACGACGGCCAGTAAGACCTTACCTGGCTGGAATC*; R: *CAGGAAACAGCTATGACCACTGTGGTTTCTCATGAGCCCTAT*) were synthesized by IDT (Integrated DNA Technologies, CA). Total 25 µL PCR reaction including 15 ng gDNA, 3 µmol/L forward and reverse M13‐tailed primer, and 1× BigDye Direct PCR Master Mix (Thermo Fisher Scientific) was incubated with PCR conditions described in BigDye™ Terminator v3.1 Cycle Sequencing Kit User Guide. Cycle sequencing was then performed using 2 µL BigDye Direct Sequencing Master Mix and 1 µL M13 forward primer or 1 µL M13 reverse primer in two separate sequencing reactions. Cycling PCR condition was applied according to the manufacturer's recommendation. Cycle‐sequencing reactions were purified using SAM/XTerminator Solution Mix (Thermo Fisher). The reaction plate was then loaded to Applied Biosystems Genetic Analyzer 3500 × L (Thermo Fisher) for capillary electrophoresis or Sanger sequencing. Post electrophoresis was completed; the ABI file of each sample was analyzed using Mutation Surveyor software (Softgenetics^®^). Sanger sequencing confirmed the variant and zygosity.

## 3 CASE REPORT

**Figure 1:**
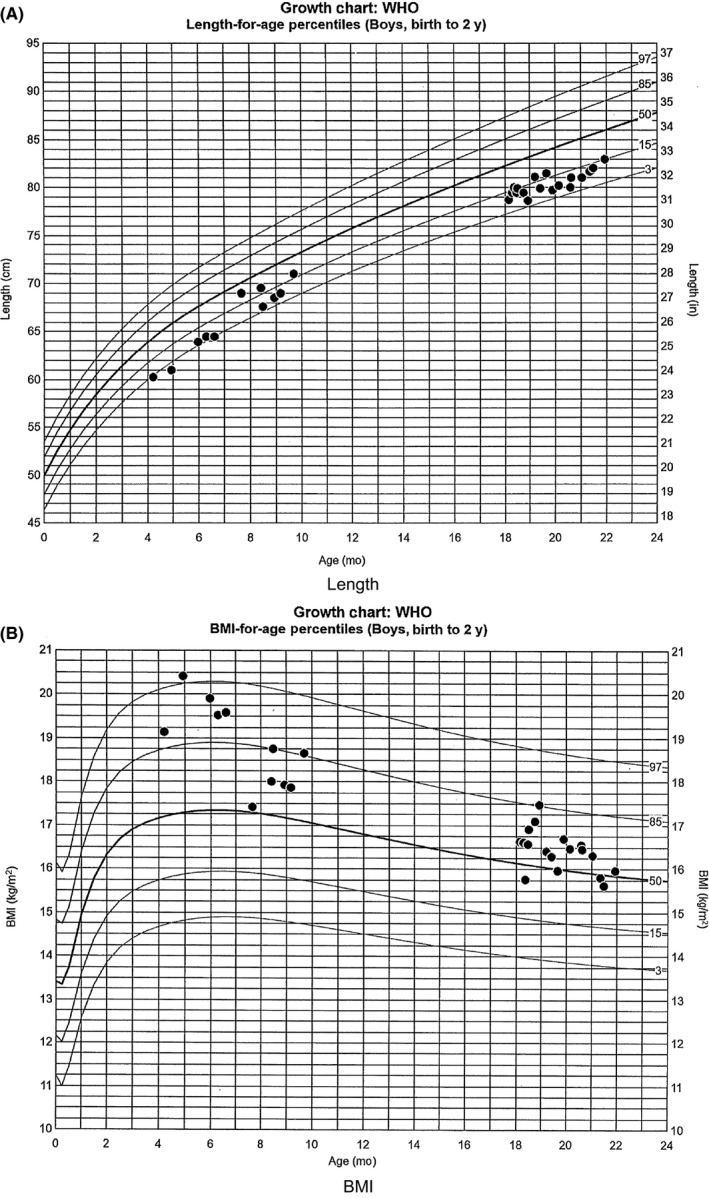
Growth chart. A, Length. B, Body mass index (BMI)

A 4‐month‐old male Hispanic infant presented with diabetes mellitus without ketoacidosis to our institution. Laboratory investigations at presentation included serum glucose 603 mg/dL, bicarbonate 17 mEq/L, and venous pH 7.325. Hemoglobin A1c at time of diagnosis was 7.2% (nondiabetic < 6.5%). GAD (glutamic acid decarboxylase) and islet cell ICA512 antibodies were negative although insulin autoantibody was markedly elevated at >50 U/mL (<0.4). He was found to have thyroid peroxidase (TPO) antibodies 81 IU/mL (normal < 9), but normal thyroid function. His mother did not have TPO antibodies indicating that their presence in this patient was not due to transplacental transfer. He was overweight with body mass index (BMI) percentile for age and gender at close to 90th percentile (Figure 1). There was no evidence of dermatitis or enteropathy. Family history was negative for known autoimmune disease, and the patient had a healthy older male sibling. There was, however, an extensive history of miscarriages on the maternal side of the family with the mother having 2 first trimester miscarriages herself. Due to the suspicious findings of both antibody‐positive diabetes mellitus and autoimmune thyroiditis in this young infant, rapid trio, whole‐genome sequencing (WGS) was performed at 5.5 months of age which identified a maternally inherited, hemizygous, pathogenic canonical splice site variant (c.−23 + 1G > T) in *FOXP3*, which was clinically confirmed by Sanger sequencing. This variant has been previously described to be causative for IPEX in 2 patients.^7^ FOXP3 protein expression analysis confirmed decreased FOXP3 expression in T regulatory cells at 25% (normal 55%‐81%) and low normal absolute FOXP3 47 cells/mcL (normal 44‐414).

After diagnosis, an IgE level was obtained which was elevated at 649 kU/L (normal 0‐8), which was consistent with the diagnosis of IPEX.^8^ Shortly thereafter, he did develop bloody stools, milk protein intolerance, eczema, and seborrheic dermatitis. Rituximab was given weekly for four doses starting at age 7 months with subsequent IVIG. Endoscopy at 9 months showed chronic inflammation, and tacrolimus was initiated without significant improvement in his symptoms.

He was seen by the blood and marrow transplant team at our institution with HLA typing and for initiation of donor search and acquisition. Decision was made to perform an allogeneic hematopoietic stem cell transplant (HSCT) at Seattle Children's Hospital, and this was performed at the age of 1 year utilizing a 10/10 HLA‐matched peripheral blood stem cell donor and a reduced intensity conditioning regimen including Treosulfan (42 g/m^2^), Fludarabine (150 mg/m^2^), and rabbit ATG (6 mg/kg). Since the transplant, his skin lesions and GI symptoms have resolved. He received regular IVIG to maintain trough serum IgG levels > 800 until it was no longer needed, 9 months post‐transplant. He had no significant infectious complications. He had mixed donor chimerism with falling donor CD3 chimerism. In an attempt to better stabilize his donor chimerism, mycophenolate was added to tacrolimus for graft vs host disease (GVHD) prophylaxis/immune suppression and the tacrolimus was subsequently discontinued in favor of sirolimus. He had no significant issues with GVHD. He is currently on a Sirolimus taper expected to be off by age 2. Over the past 3 months, he is maintaining between 83% and 86% T regulatory cells that are from the donor.

His diabetes prior to the transplant has been well controlled with multiple insulin injections using dilute lispro insulin and NPH in the morning and insulin glargine in the evening, with an A1c = 7%. NPH helped avoid extra injections during the day when he was feeding frequently and therefore not at a high risk for hypoglycemia. He was tried on a continuous subcutaneous insulin infusion via a pump using undiluted insulin when in Seattle at age 14 months, but he was not well controlled, with A1c = 7.6% and wide variability in his glucose levels, so prior to his move back to San Diego, he was switched back to multiple daily injections with diluted lispro insulin to cover for his meals, and insulin glargine as his basal insulin. His most recent A1c = 7.9% and his regimen were adjusted. We plan to have him go back on an insulin pump when he is a little bit older, once the parents are in agreement with that option, which should allow for better glycemic control. He is now 22 months old and continues to have normal thyroid function and CBC (complete blood count) without neutropenia, anemia, or thrombocytopenia as well as normal liver enzymes. At the age of 18 months, his TSH = 1.89 uIU/mL, and using a different assay (performed at the hospital's laboratory), his TPO = 16.13 IU/ml (>5.61), which seems lower compared with his initial titer that was 9 times the upper limit of normal, using a different assay. His BMI is currently close to the 50% and his length up from the 7% prior to the transplant and up to the 15% at present (Figure 1).

## 4 DISCUSSION

IPEX syndrome results from a mutation with the FOXP3 gene causing severe deficiency or absence of T regulatory cells that leads to polyendocrinopathy as well as other autoimmune manifestations and failure to thrive.^5^ As shown in this patient who developed gastrointestinal and dermatological symptoms shortly after diagnosis of diabetes mellitus, the progression of the disease can be quite quick with new manifestations occurring in rapid succession. The prognosis of IPEX is poor with most patients dying by age 2 years.^9^ Supportive treatment has primarily included hormone replacement, blood transfusions as needed for cytopenias, and immunosuppressive medications. Successful HSCT is the only known curative therapy providing for the restoration of normal FOXP3 expression and T regulatory cell function.^5^, ^10^, ^11^ The timing of HSCT is crucial, with earlier transplantation resulting in better outcomes. Thus, rapid diagnosis is key to improving morbidity and mortality. While many manifestations of IPEX are reversible following transplantation, the diabetes is generally not reversible.^5^, ^10^ We recommend that IPEX should be considered in the differential diagnosis of neonatal diabetes (diagnosis prior to age 6 months) even in the absence of other typical features of IPEX syndrome. We have the luxury in our institution of being able to perform WGS on selected patients with a very rapid turnaround time, which is why we opted for the WGS. In general, patients with diabetes diagnosed under age 6 months would warrant a neonatal diabetes panel as the initial molecular genetic testing offered, making sure that the panel includes testing for *FOXP3*. In the case presented, although the infant did not have other manifestations of IPEX, the fact that he is a male and had elevated insulin autoantibodies and thyroid antibodies should have caused us to suspect IPEX and test for *FOXP3*. As noted by Tan et al, compared with single‐gene testing for *FOXP3*, multigene panel that includes *FOXP3* and other genes of interest (see differential in that review) is most likely to identify the genetic cause of the condition at the most reasonable cost. When the phenotype is not that clear, a whole‐genome or whole‐exome sequencing 1, 12 is a useful approach in identifying the cause of neonatal diabetes before other features are present, allowing for a directed and timely intervention that may improve outcomes.

## CONFLICT OF INTEREST

Dr Ron Newfield reports grants from Merck (sitagliptin diabetes trial), and from TrialNet (NIH sponsored type 1 diabetes trials), that are outside the submitted work. The other authors declare no potential conflict of interests.

## AUTHOR CONTRIBUTIONS

SK: was the primary author and was involved in the patient's clinical care; LF, DD, SC, YD, and SK: contributed the genetic testing and interpretation and reviewed the manuscript; EJA: involved in the patient's clinical care and reviewed the manuscript; RSN: involved in the patient's clinical care and reviewed and edited the manuscript.

## References

[1] De Franco E , Flanagan SE , Houghton JA , et al. The effect of early, comprehensive genomic testing on clinical care in neonatal diabetes: an international cohort study. Lancet. 2015;386(9997):957‐963. 26231457 10.1016/S0140-6736(15)60098-8 PMC4772451

[2] Immune dysregulation, polyendocrinopathy, enteropathy, X‐linked syndrome: National Library of Medicine (US) . Genetics Home Reference [Internet]. Bethesda, MD: The Library; 2013 https://ghr.nlm.nih.gov/condition/immune-dysregulation-polyendocrinopathy-enteropathy-x-linked-syndrome

[3] Rubio‐Cabezas O , Minton JA , Caswell R , et al. Clinical heterogeneity in patients with FOXP3 mutations presenting with permanent neonatal diabetes. Diabetes Care. 2009;32:111‐116. 18931102 10.2337/dc08-1188 PMC2606841

[4] van der Vliet HJ , Nieuwenhuis EE . IPEX as a result of mutations in FOXP3. Clin Dev Immunol. 2007;2007:89017 10.1155/2007/89017. 18317533 PMC2248278

[5] Barzaghi F , Amaya Hernandez LC , Neven B , et al. Long‐term follow‐up of IPEX syndrome patients after different therapeutic strategies: An international multicenter retrospective study. J Allergy Clin Immunol. 2017;141(3):1036‐1049.e5. 29241729 10.1016/j.jaci.2017.10.041 PMC6050203

[6] Richards S , Aziz N , Bale S , et al. Standard and guidelines for the interpretation of sequence variants: a joint consensus recommendation of the American College of Medical Genetics and Genomics and the Associate for Molecular Pathology. Genet Med. 2015;17:405‐424. 25741868 10.1038/gim.2015.30 PMC4544753

[7] Otsubo K , Kanegane H , Kamachi Y , et al. Identification of FOXP3‐negative regulatory T‐like (CD4(+)CD25(+), CD127(low)) cells in patients with immune dysregulation, polyendocrinopathy, enteropathy, X‐linked syndrome. Clin Immunol. 2011;141:111‐120. 21802372 10.1016/j.clim.2011.06.006

[8] Halabi‐Tawil M , Ruemmele FM , Fraitag S , et al. Cutaneous manifestations of immune dysregulation, polyendocrinopathy, enteropathy, X‐linked (IPEX) syndrome. Br J Dermatol. 2009;160:645‐651. 18795917 10.1111/j.1365-2133.2008.08835.x

[9] Gambineri E , Perroni L , Passerini L , et al. Clinical and molecular profile of a new series of patients with immune dysregulation, polyendocrinopathy, enteropathy, X‐linked syndrome: inconsistent correlation between forkhead box protein 3 expression and disease severity. J Allergy Clin Immunol. 2008;122:1105‐1112. 18951619 10.1016/j.jaci.2008.09.027

[10] Burroughs LM , Torgerson TR , Storb R , et al. Stable hematopoietic cell engraftment after low‐intensity nonmyeloablative conditioning in patients with immune dysregulation, polyendocrinopathy, enteropathy, X‐linked syndrome. J Allergy Clin Immunol. 2010;126:1000‐1005. 20643476 10.1016/j.jaci.2010.05.021 PMC2962731

[11] Baud O , Goulet O , Canioni D , et al. Treatment of the immune dysregulation, polyendocrinopathy, enteropathy, X‐linked syndrome (IPEX) by allogeneic bone marrow transplantation. N Engl J Med. 2001;344:1758‐1762. 11396442 10.1056/NEJM200106073442304

[12] Tan Q , Louie RJ , Sleasman JW . IPEX Syndrome In: Adam MP, Ardinger HH, Pagon RA et al., eds. GeneReviews® [Internet]. Seattle, WA: University of Washington, Seattle; 1993‐2019. 20301297

